# Improving Terrestrial Squamate Surveys with Camera-Trap Programming and Hardware Modifications

**DOI:** 10.3390/ani9060388

**Published:** 2019-06-25

**Authors:** D. J. Welbourne, A. W. Claridge, D. J. Paull, F. Ford

**Affiliations:** 1 Department of Wildlife Ecology and Conservation, University of Florida, Gainesville, FL 32603, USA; 2 School of Science, University of New South Wales, Canberra, ACT 2601, Australia; andrew.claridge@environment.nsw.gov.au (A.W.C.); d.paull@adfa.edu.au (D.J.P.); 3 Office of Environment and Heritage, National Parks and Wildlife Service, Nature Conservation Section, Queanbeyan, NSW 2620, Australia; 4 Estate and Infrastructure Group, Department of Defence, Canberra, ACT 2609, Australia; frederick.ford@defence.gov.au

**Keywords:** Camera, trap, terrestrial, fauna, squamate, survey, programming, methods

## Abstract

**Simple Summary:**

Camera-traps are a useful tool in wildlife research and management, as they allow researchers to observe wildlife with minimal intrusion. However, despite their utility, the rapid pace of technological change and the relative novelty of camera-traps in wildlife research mean that many aspects of their use have not been resolved. Put simply, it may be possible to use camera-traps far more effectively than they are currently being used. Here, we examined whether detections of terrestrial snakes and lizards could be improved with camera-traps by using both time-lapse and passive infrared triggers, which are present on most camera-traps, and by adjusting the focal length of the camera to improve image clarity. Additionally, we examined whether increasing the sensitivity of passive infrared sensors is of benefit. We found that using both types of trigger simultaneously improves detections of terrestrial snakes and lizards, and users can modify the focal length to improve image clarity of fauna that occur close to the lens. These minor adjustments in the use of camera-traps result in major improvements for detecting terrestrial snakes and lizards.

**Abstract:**

Camera-traps are used widely around the world to census a range of vertebrate fauna, particularly mammals but also other groups including birds, as well as snakes and lizards (squamates). In an attempt to improve the reliability of camera-traps for censusing squamates, we examined whether programming options involving time lapse capture of images increased detections. This was compared to detections by camera-traps set to trigger by the standard passive infrared sensor setting (PIR), and camera-traps set to take images using time lapse in combination with PIR. We also examined the effect of camera trap focal length on the ability to tell different species of small squamate apart. In a series of side-by-side field comparisons, camera-traps programmed to take images at standard intervals, as well as through routine triggering of the PIR, captured more images of squamates than camera-traps using the PIR sensor setting alone or time lapse alone. Similarly, camera traps with their lens focal length set at closer distances improved our ability to discriminate species of small squamates. With these minor alterations to camera-trap programming and hardware, the quantity and quality of squamate detections was markedly better. These gains provide a platform for exploring other aspects of camera-trapping for squamates that might to lead to even greater survey advances, bridging the gap in knowledge of this otherwise poorly known faunal group.

## 1. Introduction

**Figure 1:**
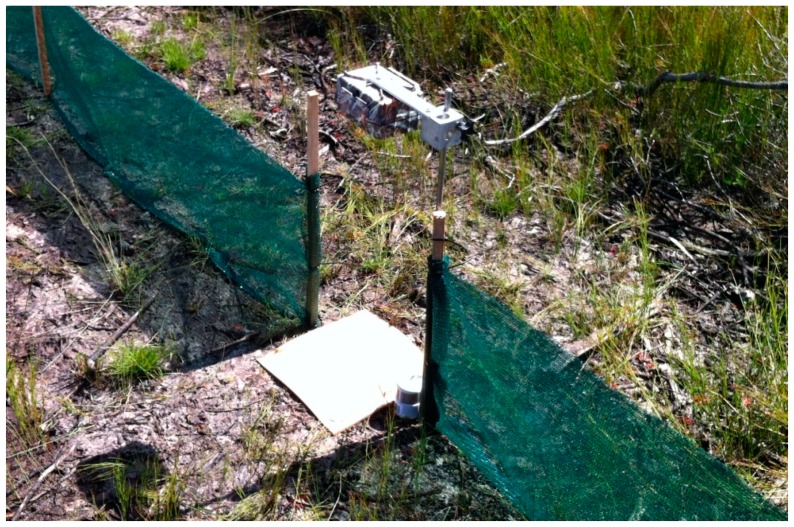
The camera overhead augmented temperature (COAT) camera-trapping method described by Welbourne [4] positions the camera-trap above a focal area that squamates are directed to by drift fences. The cork tile within the detection zone augments the thermal environment and provides the necessary thermal contrast between the target species and the background to generate detections of ectothermic species.

Camera-traps have become an increasingly useful tool for detecting various vertebrates and answering a range of research questions related to their distribution and ecology [1,2]. Although used mostly to detect mammals and birds, the utility of camera-traps has recently expanded to include detection of reptiles generally, and snakes and lizards (hereafter squamates) specifically [3]. Of most relevance, Welbourne [4] described the camera overhead augmented temperature (COAT) method for using passive infrared (PIR) triggered camera-traps to detect squamates (Figure 1), which several authors have since found to be effective to varying degrees [5,6,7,8]. However, little work has since explored how to improve the initial COAT design to make the approach more universally reliable. While there are numerous approaches to improving the method, here, we draw on experiences gained during the design of the COAT method to inform three experiments aimed at further improving squamate survey outcomes with camera-traps. Specifically, this paper assesses: (1) whether detections can be improved using the time-lapse trigger in conjunction with the PIR trigger; (2) the effectiveness of a more sensitive PIR sensor; and (3) whether altering the camera’s focal distance improves squamate identification. The motivation for these experiments follows.

### 1.1. Time-Lapse and Passive Infrared Triggers

Most modern camera-traps can generate detections of fauna with both PIR and time-lapse triggers simultaneously. Several studies have used time-lapse triggers alone to detect squamates at known refuge locations with varying success [5,9,10,11,12]. Pagnucco et al. [13] targeted the nocturnal long-toed salamander (*Ambystoma macrodactylum*) by mounting the camera-trap <0.5 m above the ground and used the PIR and time-lapse trigger simultaneously. They found the time-lapse trigger detected more amphibians than the PIR trigger and concluded that camera-traps “represent a valuable new tool for amphibian monitoring” [13] (p. 284). Since PIR triggers require a thermal contrast between the target and the background [14], using both sensors together may similarly improve squamate detections overall.

### 1.2. A More Sensitive PIR Sensor

Camera-traps are not perfect detectors as some fauna entering a camera-trap’s detection zone may go undetected [15]. Sensitivity of the PIR sensor is one feature of the camera-trap that potentially alters their responsiveness, influencing detections. Wellington et al. [16] found Reconyx HC600 camera-traps set to medium-high sensitivity detected fewer small mammals (<500 g) than when set to high sensitivity. Thus, detections with the COAT method might be improved by using a more sensitive PIR sensor. It is generally recommended to use the highest sensitivity setting when using camera-traps to detect squamates [6]; yet, Reconyx offers a high-sensitivity ‘small-mammal’ sensor, which they claim is more sensitive than the standard PIR sensor [17]. Experimenting with Reconyx camera-traps with improved sensitivity is preferable to comparing different camera-trap brands in the current study. Camera-traps certainly vary in performance between brands and even between models within the same brand [16,18,19,20,21,22]. Differences in performance manifest due to differences in PIR trigger hardware, Fresnel lens design, and software [23]. By comparing Reconyx camera-traps with varying levels of sensitivity, rather than different brands, we can make inferences about how sensitivity affects detections directly. Still, Reconyx camera-traps may not be ideal for the COAT method, or even for detecting terrestrial squamates, and another camera-trap brand might yield better results. Alternative camera-trap brands should be examined in the future.

### 1.3. In Focus

Commercially produced camera-traps are designed to capture images of target fauna that are often a metre to several metres in front of the camera. Consequently, using camera-traps in the manner described by Welbourne [4] result in blurry images since the camera-trap is placed ~70 cm from the target area, yet the focal length of the camera is >70 cm, at least with Reconyx [24] camera-traps. For many species, such images are sufficient for practitioners to identify the organism to species level, but where species are small, especially where the site is occupied by similar looking sympatric species, differentiation may not be possible. Welbourne [4] found that the grass skink (*Lampropholis guichenoti*) could not be differentiated from the delicate skink (*L. delicata*) from camera-trap images, despite being able to differentiate these species by sight [25]. Reconyx can alter the focal distance of their professional series of camera-traps at the time of purchase [17], which may overcome image focus problems. This approach to adjusting focus is preferred to using camera-traps that have autofocus, since cameras with autofocus increase the time between the trigger event and picture event. Beyond manufacturers modifying the camera-trap prior to purchase, it is also possible for practitioners to modify camera-traps themselves, as we demonstrate.

## 2. Materials and Methods

### 2.1. Study Design

To achieve the aims of this paper, three field and one laboratory-based experiment was conducted. All field experiments were conducted on Beecroft Weapons Range (BWR), ~135 km south of Sydney, Australia. The area has a temperate climate, characterised by warm summers and mild winters, and BWR is dominated by coastal heath from less than a metre to above two metres in height (see Welbourne et al. [8] for further site details). Deployment of equipment across BWR was restricted to 40 transects (~2 × ~100 m) that had vegetation cleared to understory level. Due to site access and equipment limitations, not all transects were used for each field experiment.

### 2.2. Camera-Trap Programming

Two field experiments were conducted to examine whether time-lapse triggering affects squamate detections. Two experiments were required due to a high failure rate of camera-traps in the first experiment. In the first experiment, 40 Reconyx HC600 camera-traps were deployed across 10 transects on Beecroft Weapons Range (BWR) between 16 December 2013 and 13 February 2014. Each transect consisted of four camera-trap stations positioned at 20, 40, 60, and 80 m along the transect. Each station consisted of a camera-trap, drift fence, and cork tile setup as depicted in Figure 1 and described in Welbourne [4]. The PIR trigger was set to the highest sensitivity, and the time-lapse trigger was set to capture an image every minute between 0700–1900 h. Upon completion, camera-traps remained in place and the second experiment was conducted between 14 February and 26 March 2014, with the time-lapse trigger programmed to capture an image every five minutes between 0700–1900 h. The time-lapse trigger was only used during daylight hours in both experiments since the majority of squamate species on BWR are diurnal.

Programming camera-traps to use both PIR and time-lapse triggers simultaneously meant comparisons could be made between detections from several trigger mechanism scenarios (Table 1). Comparisons were made between detections originating from: the PIR trigger only; 1-min, 5-min, or 10-min interval time-lapse trigger only; and 1-min, 5-min, or 10-min interval unique detections regardless of trigger mechanism. The longer interval detections were achieved by subsampling the 1-min interval data in the first experiment and 5-min interval data in the second experiment. Since the PIR trigger ostensibly operates continuously, the detection event was determined by the interval of the time-lapse trigger. For example, a comparison between the PIR trigger and 5-min time-lapse trigger meant the PIR trigger detection event was also five minutes; meaning that although a species may trigger the camera-trap more than once within five minutes, it was only recorded as a single event. 

We examined the effectiveness of trigger mechanism scenarios with Bayesian methods, following techniques outlined by McCarthy [26], by estimating the proportion (*p_i_*) of detections for the *i*th trigger mechanism scenario of a given squamate group (i.e., small lizards whereby snout-vent length (SVL) ≤ 50 mm); medium-large lizards (SVL > 50 mm); and, snakes). We assumed a multinomial distribution for detection frequencies (*f_i_*) and proportions were sampled from a uniform Dirichlet prior following:

(1) $$f_{i}\;\sim \;Multi\left(p_{i},\overset{i=n}{\underset{i=1}{\sum }}f\right),$$

(2) $$p_{i}\;\sim Dir\left(\alpha \right).$$

### 2.3. Camera-Trap Modification

One field and one laboratory-based experiment was conducted to examine how PIR sensor sensitivity affects squamate detections, and whether focal issues could be resolved. Experiments were conducted with 12 standard Reconyx HC600 and 12 custom modified Reconyx PC900 camera-traps. Reconyx PC900 camera-traps were modified by Reconyx before purchase in two ways: first, the focal length was adjusted to ~65 cm from the normal ~300 cm; and second, the high-sensitivity PIR sensor was installed in place of the standard PIR sensor. In all other respects, the two different models of camera-trap were identical. Given the limited number of camera-traps being compared, camera-traps were deployed for 60 days between 22 September and 22 November 2014 to increase detection histories.

**Figure 2:**
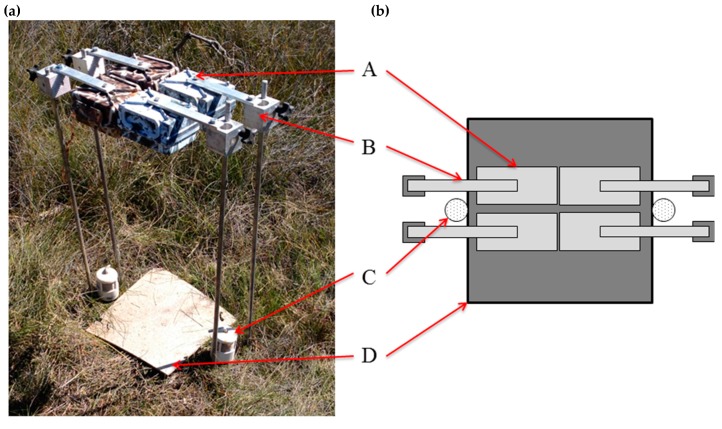
Panel (**a**) photo and (**b**) plan view of camera-trap station setup used to test focal length and passive infrared (PIR) sensor sensitivity. Two camera-traps are modified Reconyx PC900 models and two are standard Reconyx HC600 models. The setup includes: A, camera-trap; B, mounting assembly; C, bait holder baited with peanut butter and oats; and D, cork floor tile.

Six camera-trap stations were established on each of two transects (i.e., three stations per transect) on BWR. Each station consisted of four camera-traps set up as described above, but without drift fences (Figure 2). Drift fences were excluded since the camera-trap position was off the centre line of the tile and including them may have biased detections. An extra bait holder, baited with peanut butter and oats, was included to reduce the potential that animals were preferentially attracted to one side of the tile. At each station, two of the camera-traps were the modified PC900s and two were standard HC600s. Reconyx did not provide calibration data between the high-sensitivity PIR sensor and the standard PIR sensor, but suggested that the ‘Medium/High’ setting on the high-sensitivity sensor was equivalent to the ‘High’ setting on the standard sensor (J. Thinner, pers. comm. 3 Feb 2012). Consequently, at each station one camera-trap of each model was set to ‘High’ while the other was set to ‘Medium/High’ sensitivity, resulting in the following four settings being compared: standard sensor set to ‘High’ (SH); standard sensor set to ‘Medium/High’ (SMh); high-sensitivity sensor set to ‘High’ (HH); and, high-sensitivity sensor set to ‘Medium/High’ (HMh).

The effectiveness of PIR sensitivity was examined by estimating the proportions of trigger events by each sensitivity level for squamates, mammals, and false-triggers using Bayesian methods described above with uniform priors. Although mammals are not the emphasis of this paper, we included the analysis since detections of both mammals and squamates with the COAT method is possible, and to provide an indication of improved sensitivity when solely focused on mammals. Trigger frequency was used in place of detection frequency since all camera-traps at a station were sampling the same space. Thus, if all PIR sensitivities are equivalent, the proportions of the total trigger events should be equivalent between PIR sensitivity levels.

**Figure 3:**
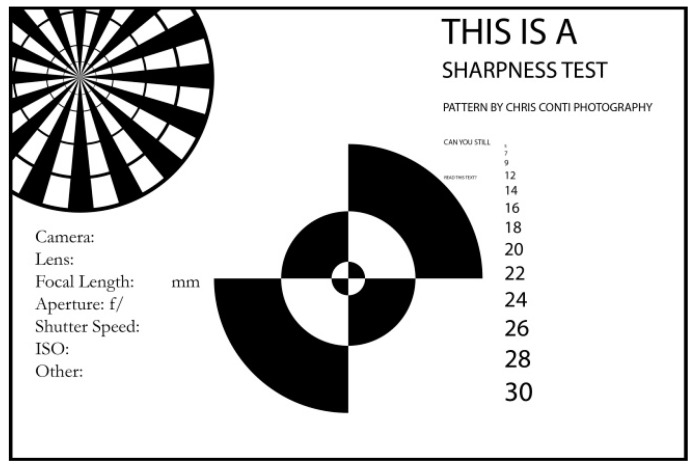
Sharpness test image used to assess focal distance of the Reconyx PC900 and Reconyx HC600 camera-traps. Source: Conti [27].

With four camera-traps focused on the same area, image clarity was simply evaluated by comparing images of the same specimen side-by-side. If the authors could identify a squamate in an image confidently, the corresponding focal length of that image was considered sufficient for identification of that species. The laboratory-based experiment examined whether the focal length could be modified by the practitioner to achieve the same results. Requiring manufacturers to adjust camera focal distance prior to purchase is not ideal. Camera-trap practitioners may have a need for camera-traps focused at the standard ~300 cm during one survey, but then in another survey a focal distance of ~65 cm might be necessary. Thus, the first author (DW) dismantled several Reconyx PC900 and HC600 camera-traps to examine hardware differences and assess whether practitioners can adjust the focal distance. Camera-traps were mounted at ~65 cm high facing a sharpness test image (Figure 3). The camera lens on the Reconyx HC600 camera-trap was then rotated to alter focal length as images were acquired. Images were then compared between the factory modified Reconyx PC900 and practitioner modified Reconyx HC600.

All analyses were conducted using *R* Version 3.1.1 [28]. To perform Bayesian analyses, the package *R2OpenBUGS* [29] was used in *R* to call *OpenBUGS* (Version 3.2.3) [30]. *OpenBUGS* software is an open source environment used to run Bayesian analyses with Markov Chain Monte Carlo (MCMC) techniques [30]. Bayesian models were run using two MCMC chains. The *R* package *CODA* was used to assess chain convergence [31]. Convergence was confirmed by visually examining convergence and autocorrelation plots, and by using Gelman and Rubin’s convergence diagnostic test to ensure shrinkage of parameter estimates were <1.05 [32]. Iterations for Bayesian models varied depending on convergence, and half of the iterations were used as burn-in. This study was conducted under the UNSW Animal Research Ethics Permits 12/14A.

## 3. Results

### 3.1. Camera-Trap Programming

Despite the number of camera-traps used and the length of the deployment in the first experiment (Dec 2013–Feb 2014), only the first 10–14 days from 16 camera-traps were useful due to a high number of failures. Failures primarily stemmed from battery failure causing camera-traps to shut down within the first week of being deployed. The cause of battery failure was not clear. Several additional camera-traps failed due to persistent false triggering, which resulted in full memory cards within several days. The remaining camera-traps operated on average for 12 days, resulting in 2486 PIR trigger events and 142,599 time-lapse trigger events. The second experiment (Feb–Mar 2014) had only three camera-trap failures, resulting in three camera-trap station pairs being removed from the analysis. The remaining 34 camera-traps (17 pairs) operated for 41 days each, resulting in 1394 camera-trap days, 9994 PIR trigger events, and 196,112 time-lapse trigger events. During the two experiments, ten squamate species were detected with varying success between trigger mechanisms (Table 2).

**Figure 4:**
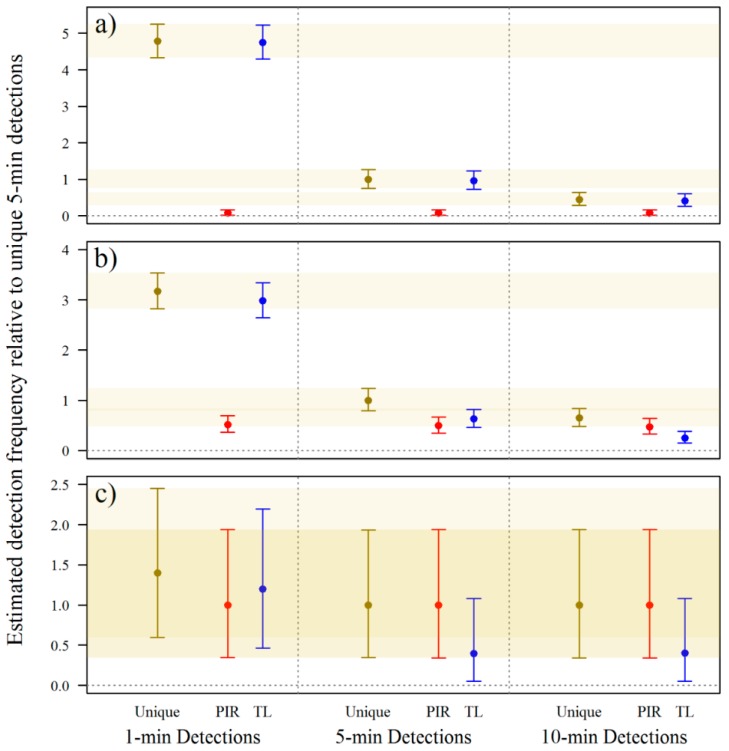
Estimates of the proportion of detection events during Dec 2013–Feb 2014 of (**a**) small lizards; (**b**) medium–large lizards; and (**c**) snakes as a function of trigger mechanism. Estimates are standardised to unique 5-min detections. Trigger mechanism scenarios are unique detections, passive infrared (PIR) trigger only detections, and time-lapse (TL) trigger only detections. Central dots represent the mean of the posterior distributions and bars represent 95% credible intervals.

**Figure 5:**
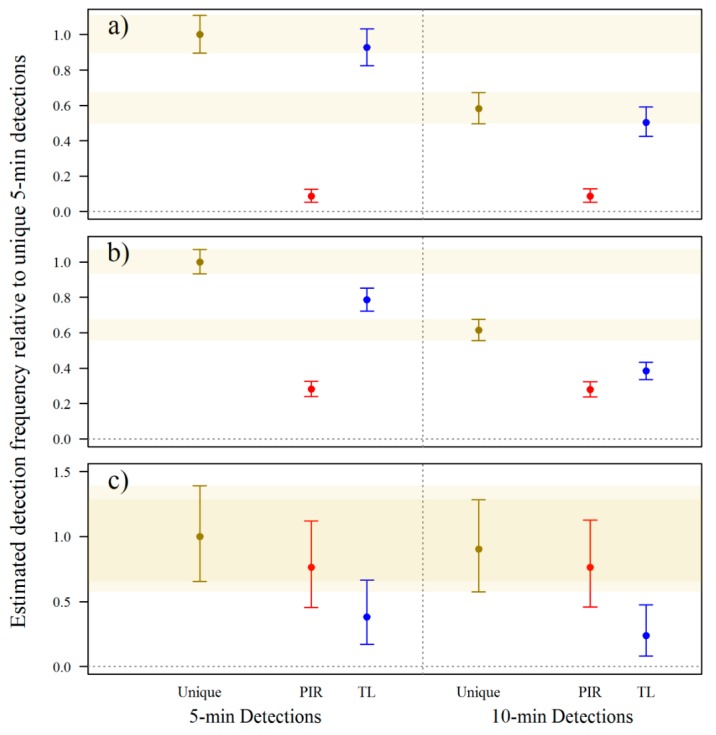
Estimated detection frequencies during the second experiment (Feb–Mar 2014) of (**a**) small lizards; (**b**) medium–large lizards; and (**c**) snakes as a function of trigger mechanism relative to unique 5-min detections. Trigger mechanism scenarios are unique detections, passive infrared (PIR) trigger only detections, and time-lapse (TL) trigger only detections. Central dots represent the mean of the posterior distributions and bars represent 95% credible intervals.

Standardised estimates of the proportions of detection events demonstrated that detection frequency was not uniform between trigger mechanism, in both the first (Figure 4) and second experiment (Figure 5). Shorter time-lapse intervals increased detections overall (unique) of small and medium–large lizards. The 1-min detection interval resulted in nearly five times the detections of small lizards and approximately three times the detections of medium–large lizards than the 5-min detection interval. Conversely, the 10-min detection interval resulted in only ~50–60% of the detections achieved with the 5-min detection interval for small and medium–large lizards. The evidence suggests that shorter detection intervals were no more effective than longer detection intervals for detecting snakes since the PIR trigger accounted for most detections.

Generally, time-lapse triggers resulted in more detections of small and medium–large lizards than PIR triggers. This was most apparent for small lizards where, regardless of detection interval, unique detections and time-lapse detections were ostensibly the same. Unique detections of medium–large lizards, when using 5- or 10-min detection intervals were generally higher than either time-lapse or PIR detections alone. Thus, maximum detections of medium–large lizards were achieved using both time-lapse and PIR triggers, but maximum detections of small lizards were achieved using the time-lapse trigger alone. For snakes, PIR triggers are likely more effective than time-lapse triggers, and using both triggers together did not greatly improve detections. In the first experiment, PIR and time-lapse triggers do not appear to have performed differently to each other, but in the second experiment, time-lapse triggers detected snakes on fewer occasions than PIR triggers.

### 3.2. Camera-Trap Modification

One camera-trap station was removed in the final analysis since the two Reconyx HC600 camera-traps at the station failed within the first week of being deployed. One camera-trap appeared to fail due to a battery problem and the second false-triggered continuously until the memory card was full after three days. Several other camera-traps stopped working before the recovery date due to either full memory cards or battery failure. In those cases, data from the remaining camera-traps at the station were pared back to ensure equivalent deployment periods were analysed. Still, each camera-trap operated for an average of 56 days resulting in 1120 camera-trap days total. A total of 3893 trigger events occurred during the experiment. Squamates accounted for ~4.8% (*n* = 190) of triggers, mammals ~10.2% (*n* = 397), birds ~2.7% (*n* = 104), false-triggers accounted for ~82.2% (*n* = 3201) of triggers, and there was one human triggered event. Overall, seven squamate and eight mammal species were detected (Table 3).

#### 3.2.1. Passive Infrared Sensitivity

**Figure 6:**
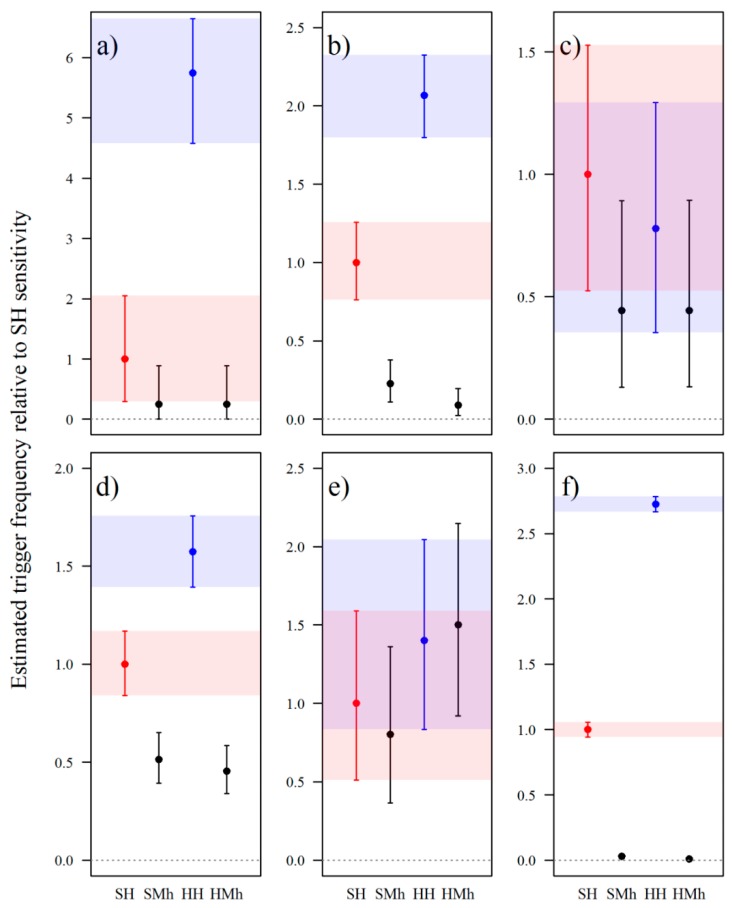
Estimated trigger frequencies as a function of passive infrared (PIR) sensor sensitivity for six groups, relative to the standard PIR sensor set to ‘High’ (SH, red). Groups are: (**a**) small lizards; (**b**) medium–large lizards; (**c**) snakes; (**d**) small mammals; (**e**) medium–large mammals; and, (**f**) false triggers. Passive infrared sensitivity levels are: SH, standard sensor set to ‘High’; SMh, standard sensor set to ‘Medium/High’; HH (blue), high-sensitivity sensor set to ‘High’; HMh, high-sensitivity sensor set to ‘Medium/High’. Central dots represent the mean of the posterior distributions and bars represent 95% credible intervals.

Standardised estimates of trigger frequencies demonstrated that trigger events were not uniform across PIR sensitivity levels for most groups (Figure 6). Passive infrared sensitivity does not appear to alter the frequency of trigger events for snakes or medium–large mammals (Figure 6c,e). Nevertheless, the wide credibility intervals in these two groups reflect the few trigger events recorded. The HH sensor was more effective than the standard high sensitivity (SH) sensor for detecting small lizards, medium–large lizards, and small mammals; triggering 5.75 (Credible Interval (CI) 4.58–6.64), 2.07 (CI 1.80–2.32), and 1.57 (CI 1.39–1.76) times more frequently, respectively. For the same faunal groups, ‘Medium/High’ sensitivity levels (i.e., SMh and HMh) triggered <50% as often as SH. Nevertheless, there may be no difference in trigger frequency between SH, SMh, and HMh sensitivities for small lizards. Although the HH sensitivity level generally triggered most frequently, it also had 2.73 (CI 2.67–2.78) times the false-triggers of SH (Figure 6f).

#### 3.2.2. Focal Length

**Figure 7:**
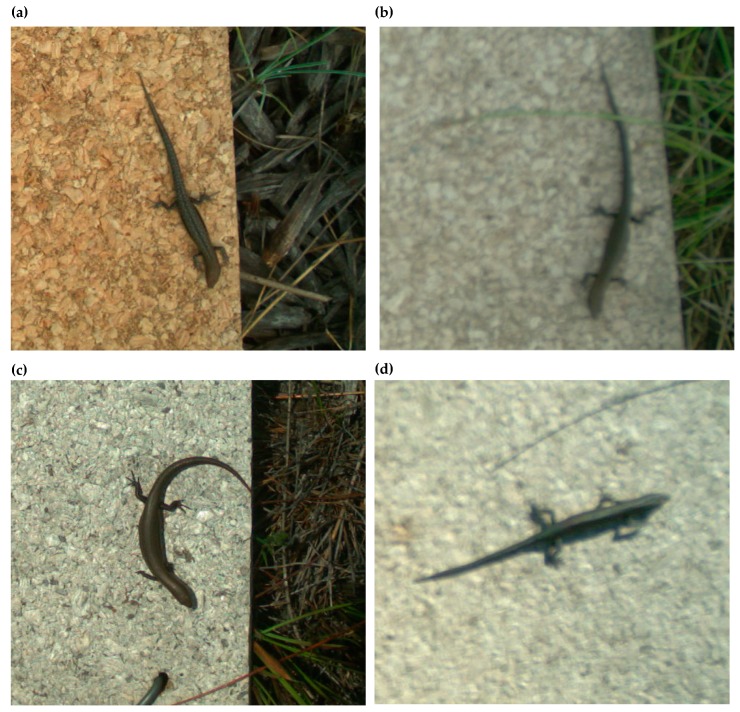

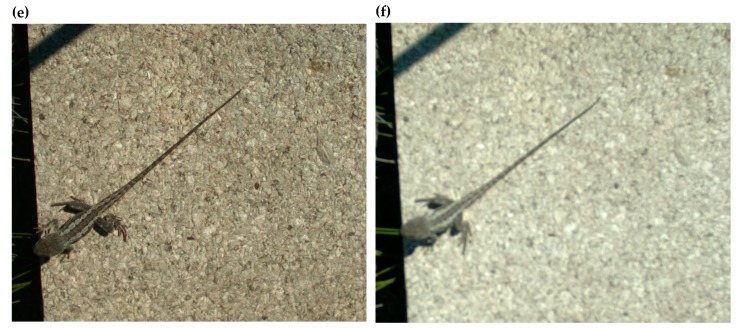
Enlarged sections of images of the same individual grass skink (*Lampropholis guichenoti*) (**a**,**b**), garden skink (*Lampropholis delicata*) (**c**,**d**), and jacky dragon (*Amphibolurus muricatus*) (**e**,**f**). Images (**a**,**c**,**e**) were captured by the modified Reconyx PC900 camera-trap and images (**b**,**d**,**f**) were captured by the standard Reconyx HC600 camera-trap.

Focal distance made a tangible difference to species identification. Both *L. delicata* and *L.* *guichenoti* were detected, but positive demarcation between these species was only possible with the modified focal distance of the Reconyx PC900 camera-traps (Figure 7). The key diagnostic feature between these species is the vertebral stripe and white flecking exhibited by *L. guichenoti*, which is absent on *L. delicata*. Images from the standard focal distance (~300 cm) were too blurry to provide definitive identification (Figure 7b,d). The superior sharpness of the modified focal distance also improved the clarity of ornamental patterns exhibited by some species, making identification of individuals far easier (Figure 7). Nevertheless, other than *Lampropholis* spp., identification of all other fauna to species-level was possible with the standard focal length camera-trap.

**Figure 8:**
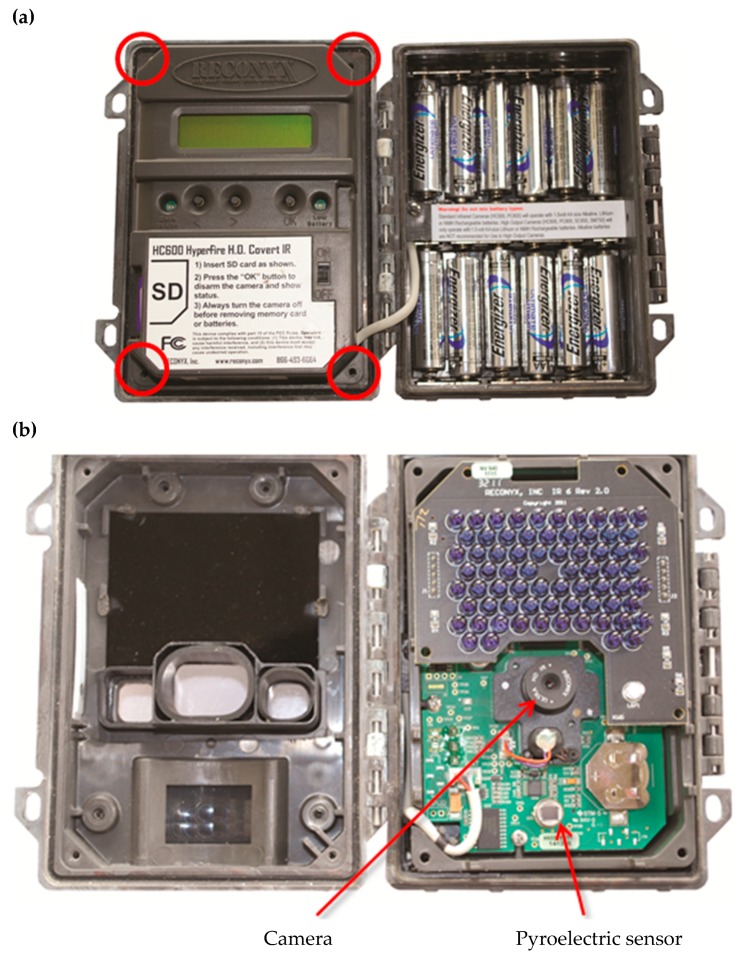
By removing the four screws highlighted in image (**a**), internal components (**b**) of the Reconyx PC900 and Reconyx HC600 camera-trap can be accessed.

**Figure 9:**
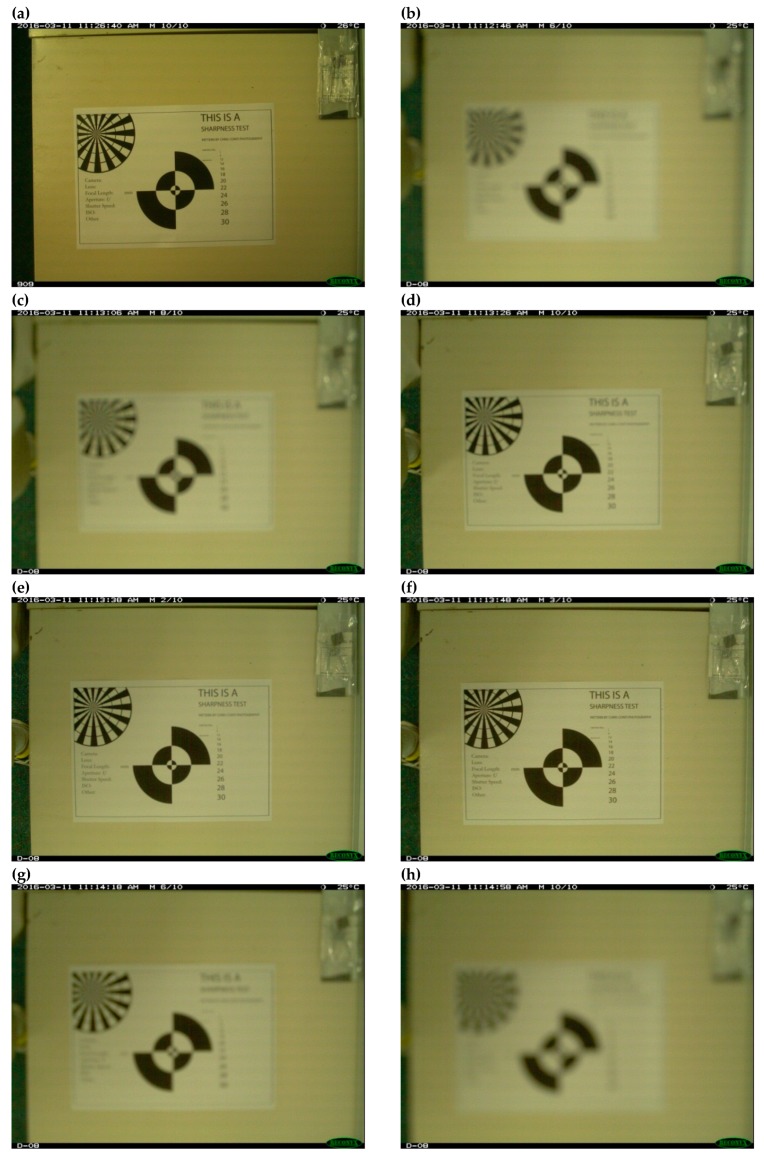
Image sharpness test results from (**a**) a factory modified Reconyx PC900, and (**b**)–(**h**) a practitioner modified Reconyx HC600 camera-trap. Images (**b**)–(**h**) show the change in image sharpness as the camera lens is wound through its focal range.

It was possible for the authors to modify the focal distance of the standard Reconyx HC600 camera-trap without specialist tools. Although the process voids the warranty (Reconyx, 2010), accessing the camera within the camera-trap is accomplished by removing four screws from inside the camera-trap housing (Figure 8a). The camera of the Reconyx PC900 and HC600 are the same make and model. By winding the camera lens of the Reconyx HC600 through its range, the focal distance changed (Figure 9). The sharpness test image, which was clear with the factory modified Reconyx PC900, was initially blurry with the unmodified Reconyx HC600. Winding the camera lens through its range sharpened the image, comparable to the Reconyx PC900, and then became blurry again as the focal distance became too short.

## 4. Discussion

Developing new, and enhancing existing survey methods in wildlife research and management is necessary to overcome data paucity. This is especially apparent for squamates because, despite being one of most speciose groups of terrestrial vertebrates, compared with mammals or birds [33], little is understood about the roles squamates play in ecological systems or their threat status. For example, the IUCN [34] has been unable to provide an estimate of the proportion of reptiles that are threatened globally due to data deficiency. Here, we examined whether detections of squamates with existing camera-trap techniques, specifically the COAT method, could be improved using time-lapse triggers, more sensitive PIR sensors, or by modifying the camera-trap’s focal distance. We found time-lapse triggers greatly contribute to detections of small- and medium-sized lizards, and using both PIR and time-lapse triggers simultaneously is most effective generally. Increasing the sensitivity of the PIR sensor does improve detections overall, especially of smaller species, but generally this is not a good solution for practitioners. Finally, camera-trap focal distance, at least in Reconyx camera-traps, can be modified by the practitioner and is necessary for identifying small, similar looking sympatric species.

For effective squamate detection, there is no question that the time-lapse trigger, set at 5-min detection intervals, should be used in conjunction with the PIR trigger to improve data collection. Using either trigger alone is not ideal since detections of certain squamate groups with only a single detector were compromised. If a particular group is the focus of a study (e.g., snakes only), then using the trigger most suitable to that group would suffice. Using time-lapse at 1-min detection intervals would improve detections further, and may obviate the need for PIR sensors entirely, but doing so requires regular camera-trap maintenance [10,13]. In fact, the failures observed in the first experiment were almost certainly attributable to the high frequency of image acquisition. Technological developments may remove the need for regular maintenance. For example, increased memory capacity can be achieved with high capacity memory cards, or by using camera-traps that offer remote download functionality [35]. Additionally, solar panels can be combined with camera-traps to charge batteries and reduce battery maintenance [36]. False-trigger events, or images without fauna do present an issue for data processing and storage, but developments in automated identification processes will likely remove many of these problems [37].

Custom modification of camera-traps can improve results, but practitioners will need to evaluate their circumstances and research questions carefully before adopting such tools. The modification to the high-sensitivity PIR sensor shown here was essentially a one-way process [38]. As such, the utility of the modified camera-traps was greatly reduced since, unless used on the medium-high sensitivity setting, it is not possible to compare data from the modified camera-traps with standard camera-traps without extensive calibration. The high-sensitivity sensor clearly increased detections of squamates and small mammals, since small species exhibit smaller thermal signatures, but they were no more effective than the standard sensor for larger species. Given the results of using time-lapse triggers with the PIR sensor, the optimal solution for squamate surveys at this point seems to be simply to use the standard PIR sensor and time-lapse triggers simultaneously.

Modifying the focal length of the camera-trap is desirable as it provided clearer images of small squamates. Sharp images allowed for differentiation between particular sympatric species (i.e., *Lampropholis* spp.), which overcame a key limitation observed in other studies [6,8]. Additionally, sharper images likely permit researchers to derive certain morphological traits of squamates, such as head size or SVL, in a similar manner to how morphological traits of fish are collected with camera-traps [39]. Modifying the focal length does not diminish the utility of the camera-trap since the modification can be completed and reversed by the practitioner. Still, practitioners need to evaluate whether this is worthwhile for their circumstances since, regardless of image clarity, some species may only be differentiable by features exhibited on ventral or lateral surfaces [25]. Reconyx [24] state that opening the inner case of their camera-trap voids the associated warranty. Therefore, perhaps the more pertinent issue is that manufacturers need to allow practitioners to conduct simple modifications. Other camera-trap manufacturers, for example Bushnell, are fulfilling this need for practitioners to adjust focal length by providing different lenses [40].

## 5. Conclusions

The three components tested here to improve detections of squamates with camera-traps are but a limited set of aspects of camera-trapping that demand further attention. Improvements in fauna detections generally, and squamates specifically may come from examining numerous other aspects of how camera-traps are used. These characteristics, however, appeared most fruitful during the development of the COAT method. Although including time-lapse with PIR triggers and modifying the focal length improved squamate detection results in the present study, these results may not necessarily generalise to other systems. Practitioners should undertake initial experimentation before committing to a survey method or adopting a new method, as no survey technique is a panacea for fauna detection.

## Figures and Tables

**Table 1:** Unique, passive infrared (PIR), and time-lapse (TL) trigger mechanism scenarios used to detect squamates during two field experiments.

Trigger Mechanism	Detection Description
Experiment 1 (Dec 2013–Feb 2014)	
Uni 1	Unique detections with 1-min TL intervals.
Uni 5	Unique detections with 5-min TL intervals.
Uni 10	Unique detections with 10-min TL intervals.
TL 1	TL detections only with 1-min TL intervals.
TL 5	TL detections only with 5-min TL intervals.
TL 10	TL detections only with 10-min TL intervals.
PIR	PIR detections only matched to interval
Experiment 2 (Feb–Mar 2014)	
Uni 5	Unique detections with 5-min TL intervals.
Uni 10	Unique detections with 10-min TL intervals.
TL 5	TL detections only with 5-min TL intervals.
TL 10	TL detections only with 10-min TL intervals.
PIR	PIR detections only matched to interval

**Table 2:** Squamate species detected with either passive infrared (PIR) or time-lapse (TL) trigger for 1-, 5-, and 10-min detection intervals.

Scientific Name	Common Name	PIR	Time-Lapse		
			1-min	5-min	10-min
Small lizards (SVL ≤ 50 mm)					
*Lampropholis* spp.^a^	Delicate and grass skink	*	*	*	*
*Saproscincus mustelinus*	Weasel skink	*	*	*	
Medium–large lizards (SVL > 50 mm)					
*Acritoscincus platynota*	Red-throated skink	*			
*Amphibolurus muricatus*	Jacky dragon	*	*	*	*
*Ctenotus taeniolatus*	Copper-tailed skink	*	*	*	*
*Cyclodomorphus michaeli*	Mainland she-oak skink		*		
*Tiliqua scincoides*	Blue-tongue skink	*			
Snakes					
*Hemiaspis signata*	Marsh snake		*	*	*
*Pseudechis porphyriacus*	Red-bellied black snake	*		*	*
*Pseudonaja textilis*	Eastern brown snake	*	*	*	*

^a^*Lampropholis* spp. were not identifiable to species-level due to poor focus.

**Table 3:** Squamate and mammal species detected using both modified Reconyx PC900 and standard Reconyx HC600 camera traps. Camera traps were set to either ‘High’ or ‘Medium/High’ sensitivity levels resulting in: SH, standard sensor set to ‘High’; SMh, standard sensor set to ‘Medium/High’; HH, high-sensitivity sensor set to ‘High’; and HMh, high-sensitivity sensor set to ‘Medium/High’.

Group/Scientific Name	Common Name	Reconyx HC600		Reconyx PC900	
		SH	SMh	HH	HMh
Small lizards (SVL ≤ 50 mm)					
*Lampropholis* spp.^a^		*			
*Lampropholis delicata*	Garden skink	NA	NA	*	
*Lampropholis guichenoti*	Grass skink	NA	NA	*	
Medium–large lizards (SVL > 50 mm)					
*Amphibolurus muricatus*	Jacky dragon	*	*	*	*
*Ctenotus taeniolatus*	Copper-tailed skink	*		*	
*Cyclodomorphus michaeli*	Mainland she-oak skink			*	
Snakes					
*Pseudechis porphyriacus*	Red-bellied black snake	*	*	*	*
*Pseudonaja textilis*	Eastern brown snake	*	*	*	*
Small mammals (<500 g)					
*Antechinus stuartii*	Brown antechinus	*	*	*	*
*Cercartetus nanus*	Eastern pygmy possum			*	
*Rattus fuscipes*	Bush rat	*	*	*	*
*Rattus*	Black rat	*	*	*	*
Medium–large mammals (≥500 g)					
*Perameles nasuta*	Long-nosed bandicoot	*	*	*	*
*Macropus giganteus*	Eastern grey Kangaroo	*	*	*	*
*Tachyglossus aculeatus*	Short-beaked echidna	*	*	*	*
*Wallabia bicolor*	Black wallaby	*	*	*	*

^a^*Lampropholis* spp. were not identifiable to species-level using the standard Reconyx HC600 camera trap.
